# Synthesis of Polymer—Mesoporous Silica Nanocomposites

**DOI:** 10.3390/ma3074066

**Published:** 2010-07-13

**Authors:** Liangming Wei, Nantao Hu, Yafei Zhang

**Affiliations:** National Key Laboratory of Nano/Micro Fabrication Technology, Key Laboratory for Thin Film and Microfabrication of Ministry of Education, Institute of Micro and Nano Science and Technology, Shanghai Jiao Tong University, Dongchuan Road, Shanghai, 200240, China; E-Mails: hunantao@sjtu.edu.cn(N.T.H.); lmwei@sjtu.edu.cn(L.M.W.)

**Keywords:** mesoporous silica, polymer, nanocomposites

## Abstract

Polymer nanocomposites show unique properties combining the advantages of the inorganic nanofillers and the organic polymers. The mesoporous silica nanofillers have received much attention due to their ordered structure, high surface area and ease for functionalization of the nanopores. To accommodate macromolecules, the nanopores lead to unusually intimate interactions between the polymer and the inorganic phase, and some unusual properties can be observed, when compared with nonporous fillers. Whereas many review articles have been devoted to polymer/nonporous nanofiller nanocomposites, few review articles focus on polymer/mesoporous silica nanocomposites. This review summarizes the recent development in the methods for synthesizing polymer/mesoporous silica nanocomposites based on the papers published from 1998 to 2009, and some unique properties of these composites are also described.

## 1. Introduction 

Polymer nanocomposites are a class of hybrid materials composed of an organic polymer matrix with dispersed inorganic nanofillers. Polymer nanocomposite shows unique properties, combining the advantages of the inorganic nanofillers (e.g., rigidity, thermal stability) and the organic polymers (e.g., flexibility, dielectric, ductility, and processability) [1]. The inorganic nanofillers have large surface area, leading to a dramatic increase in interfacial area [1,2,3]. These nanofillers, even at very low concentrations, can strongly change the macroscopic properties of the polymer [4]. Inorganic nanofillers include nanotubes, metal oxides (e.g., SiO_2_, TiO_2_, Al_2_O_3_, Fe_3_O_4_), layered silicates (e.g., montmorillonite, saponite), metalic nanoparticles (e.g., Au, Cu), semiconductors (e.g., PbS, CdS) [1], and mesoporous silicas, *etc*. Whereas many review articles have been devoted to polymer/ nonporous nanofiller nanocomposites [1,2,3], few review articles focus on polymer/mesoporous silica nanocomposites. The mesoporous silica nanofillers have received much attention due to their ordered structure, high surface area and easiness for functionalization of the nanopores. The nanopores are sufficiently porous to accommodate macromolecules which will lead to unusually intimate interactions between the polymer and the inorganic phase [2], and some unusual properties will be observed awhen compared to those nonporous fillers. This review summarizes the recent developments in the methods for synthesis of polymer/mesoporous silica nanocomposites based on the papers published from 1998 to 2009. This review will show a general route for the preparation of the nanocomposites, and then give a brief discussion of the properties of the composites.

## 2. Mesoporous Silica and Surface Functionalization

The first mesoporous silica, known as the M41S phase, was developed by the Mobil Oil Company in 1992. Unlike the zeolite, the M41S materials have pore diameters from approximately 2 to 10 nm [5]. This class of material has very large surface areas, ordered pore systems, and well-defined pore radius distributions [5]. The most well-known types of this class of materials include the silica solids MCM-41 (with a hexagonal arrangement of the mesopores, space group *p6mm*), MCM-48 (with a cubic arrangement of the mesopores, space group *Ia3 d*), and MCM-50 (with a laminar structure, space group *p2*) [5]. The use of amphiphilic triblock copolymers as a structure-directing agents has resulted in the preparation of well-ordered hexagonal mesoporous silica structures (SBA-15, SBA: Santa Barbara University) with uniform pore sizes up to approximately 30 nm [5,6]. According to the definition of IUPAC, mesoporous materials are described as materials whose pore diameters lie in the range between 2 and 50 nm [5].The composites based on the microporous silica with the pore size small than 2 nm and the macroporous silica with the pore size above 50 nm are excluded from this review. 

Mesoporous silica is synthesized via polycondensation of silica species, which originate from different sources of silica (like sodium silicate, tetraethyl- (TEOS) or tetramethylorthosilica (TMOS)) in the presence of surfactants as structure-directing agents [5,6]. Many types of ionic (e.g., hexadecyltrimethylammonium bromide (CTAB)) and non-ionic surfactants (e.g., amphiphilic triblock copolymers) have been used for obtaining mesoporous silica with different pore structure and morphological characteristics [6]. To full outline the preparation of mesoporous silica, readers are referred to excellent reviews published in these years [5,6]. 

**Figure 1:**
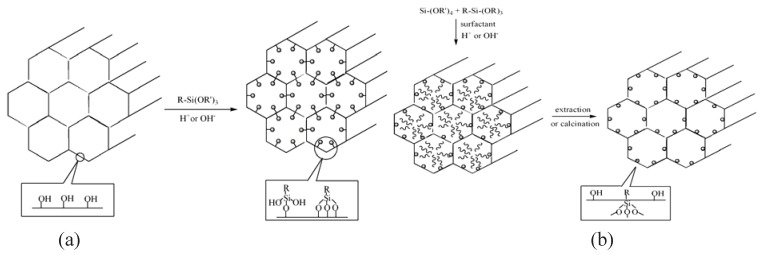
(a) Postsynthetic functionalization and (b) co-condensation methods for organic modification of mesoporous silica.

Both the dispersion of nanofillers in the polymer matrix and the interaction between nanofillers with the polymers is closely related to the properties of composites [4]. In order to get a well-dispersed composite with enhanced interfacial adhesion, surface modification of the nanoparticles is always necessary. Some typical silane coupling agents used for surface functionalization of mesoporous silica are shown in Table 1. Two main pathways (postsynthetic functionalization and co-condensation method) are available for the modification of the surface of mesoporous, whose mechanisms are shown in Figure 1. The readers are encouraged to read the review by Hoffmann *et al.* [5].

**Table 1:** Typical silane coupling agents used for surface functionalization of mesoporous silica.

Name	Structure
3-aminopropyltriethoxysilane	H_2_N(CH_2_)_3_Si(OC_2_H_5_)_3_
3-aminopropyltrimethoxysilane	H_2_N(CH_2_)_3_Si(OCH_3_)_3_
Vinyltriethoxysilane	CH_2_=CHSi(OC_2_H_5_)_3_
Vinyltrimethoxysilane	CH_2_=CHSi(OCH_3_)_3_
3-isocyanatopropyltriethoxysilane	OCN(CH_2_)_3_Si(OC_2_H_5_)_3_
methacryloxymethyltriethoxysilane	CH_2_=C(CH_3_)COO(CH_2_)_3_Si(OC_2_H_5_)_3_
3-methacryloxypropyltrimethoxysilane	CH_2_=C(CH_3_)COO(CH_2_)_3_Si(OCH_3_)_3_
mercaptopropyl triethoxysilane	SH(CH_2_)_3_Si(OC_2_H_5_)_3_
methyltriethoxysilane	CH_3_Si(OC_2_H_5_)_3_
phenyltrimethoxysilane	PhSi(OCH_3_)_3_
bis(triethoxysilylpropyl)tetrasulfane	(C_2_H_5_O)_3_Si(CH_2_)_3_S_4_(CH_2_)_3_Si(OC_2_H_5_)_3_
3-glycidoxypropyltrimethoxysilane	CH_2_(O)CHCH_2_O(CH_2_)_3_Si(OCH_3_)_3_
Dimethyldichlorosilane	(CH_3_)_2_SiCl_2_

## 3. Preparation of Polymer/Mesoporous Silica Nanocomposites

### 3.1. Blending

Direct mixing of the mesoporous silica into the polymer is the simplest method for preparing polymer/mesoporous silica composites. The mixing can be done by melt blending and solution blending [1]. However, to fully disperse nanoparticles in the polymer matrix is a big challenge due to the strong tendency of nanoparticles to agglomerate. Surface modification of the nanofillers is a general route to enhance the dispersion of the nanofillers and to build up a strong interfacial interaction between the polymer and inorganic nanofillers [1].

Melt blending is done by mixing the mesoporous silica with the polymer above the melting point or above the glass-transition temperature (Tg) of polymers. This method is efficient and operable [1]. 

Pérez *et al.* reported styrene-butadiene rubber (SBR)/mesoporous silica nanocomposites synthesized by mixture of SBR and mesoporous silica [7,8,9]. The addition of mesoporous silica into SBR led to an increase in swelling degree, elastic modulus and Tg of the samples. It was observed that the vulcanization rate was higher for the rubber filled with mesoporous silica, which can be explained by chemical interaction between the fillers and rubbers and the penetration of the rubber chains into the mesopores [7,8,9].

Wang *et al*. reported that mixture of polypropylene (PP) with Na-montmorillonite (Na-MMT) and MCM-41, generating a nanocomposite filled with two different kinds of nanoparticles. In this nanocomposite, Na-MMT layers were exfoliated and the hexagonal framework structure of MCM-41was retained. The mechanical tests showed that with simultaneous nanofiller loading, the co-incorporation of Na-MMT and MCM-41 into the PP matrix gave rise to much enhanced effects than incorporating either Na-MMT or MCM-41 particles separately, due to different interfacial structure between the fillers and the matrix in the PP composite [10].

Solution blending is done by mixing mesoporous silica and polymer in a solvent. After removal of the solvent, the composites are obtained. This method brings about a good molecular level of mixing, and can overcome the limitations of melt mixing [1]. Many polymers have a good mixture with mesoporous silica using this method. However, solution blending has some disadvantages. For example, the appropriate solvent is not easy to obtain and it is necessary to remove the solvent after processing.

Most of the PEO/mesoporous silica nanocomposites were obtained using solution blending [11,12,13,14,15,16,17]. Generally, the mesoporous silica was first dispersed in solvent (e.g., acetonitrile, acetone or methanol), and then PEO and Li^+^ ions were added to the solution. After the mixture was stirred and the solvent removed, nanocomposites filled with mesoporous silica were obtained. Functionalization of the surface by organic groups (such as amino, 3-glycidyloxypropy groups) is usually used to improve dispersion and compatibility between PEO and mesoporous silica or to promote fast ion transfer [11,12,13,14,15,16,17]. The PEO-based nanocomposites can be used for composite polymer electrolytes. Addition of mesoporous silica nanofillers into the PEO matrix led to an increase in the ionic conductivity and lithium ion transference number of the composite electrolytes.

Nafion/mesoporous silica composite films were prepared by blending Nafion resin and mesoporous silica in dimethyl formamide (DMF) [18,19]. It was found that the Nafion composites filled with mesoporous silica have approximately 1.5 times higher proton conductivities than pure Nafion, and can display good temperature performance relative to pure Nafion and the SiO_2_ particle composite [18]. 

Encapsulation of conducting polymers in the nanopores can inhibit interchain effects, resulting in unusual propertied for the polymers [20,21,22,23,24,25]. Posudievsky *et al*. reported guest–host poly[2-methoxy-5-(2′-ethyl-hexyloxy)-1,4-phenylene vinylene] (MEH-PPV)/MCM-41 composites via insertion of macromolecules inside mesoporous silica in toluene or chlorobenzene [22]. The intensity of photoluminescence of the obtained composites was found to be two orders of magnitude greater than that of the neat polymer [22]. The MEH-PPV/ MCM-41 films with highly oriented and ordered *2d* rectangular *cmm* structure were also reported by Molenkamp *et al.* [25]. Other conducting polymers, such as poly (2,8-dibenzothiophene-5,5-dioxide-vinylene)-*alt*-(2-methoxy-5-alkoxy-1,4-phenylenevinylene) (DDMA–PPVs) and poly(9,9’-dioctylfluorene) were also reported to be mixed with MCM-41 in solvent to form composites [26,27]. The results showed evidence of the molecular orbital confinement effect in the composites [26], and the resulting nanocomposite film showed dramatically increased photostability and color purity, as well as higher electroluminescence output [27].

Some studies focused on MCM-41 as an additive to enhance the gas permeability characteristics of high-performance polysulfone films. The polysulfone/mesoporous silica composite films were prepared by mixing polysulfone and mesoporous silica in solvent [28,29]. It was found that for all gases tested (N_2_, O_2_, CO_2_, CH_4_), the permeability increased in proportion to the weight percent of MCM-41 present in the film, without a loss in selectivity [28].

The poly(dimethylsiloxane)/mesoporous silica composite was prepared by Kim *et al*. [30]. This material can be loaded with the drug ibuprofen, which can be released from the system by ultrasound. The composites were prepared by mixing the ibuprofen-loaded mesoporous silica with poly(dimethylsiloxane) solution. Other siloxane polymer (e.g., poly(di-*n*-hexylsilane), poly(methylphenylsilane) based composites were also obtained using solution blending [31,32]. 

### 3.2. In Situ Polymerization

#### 3.2.1. General Polymerization

The *in situ* polymerization method has many advantages, such as ease of handling and better performance of the final products [1]. Generally, this method involves the dispersion of nanofillers in monomer(s), and then bulk or solution polymerization. The nanofillers are always modified by functional groups to increase the interaction between the polymers and the nanofillers, or to get a good dispersion in the polymer matrix. For example, as-synthesized MCM-48 was modified via silylation with Cl-Si(CH_3_)_3_ to generate hydrophobic surface [33].

In 1998, Moller *et al*. reported poly(methyl methacrylate)(PMMA)/mesoporous silica composites synthesized by adsorption of MMA into the pores and then polymerization initiated with benzoyl peroxide. It was found that the polymers confined in the 6-35 Å diameter channels of the hosts did not show characteristic bulk behavior with respect to their glass transition temperature [34]. Recently, Zhang *et al*. synthesized PMMA-mesocellular foam silica nanocomposites via batch emulsion polymerization [35]. Ji *et al*. synthesized a propyl methacrylate functionalized mesoporous silica by condensation of TEOS and (3-trimethoxysilyl)propyl methacrylate [36] in the presence of surfactants. *In situ* polymerization of (3-trimethoxysilyl)propyl methacrylate among the mesoporous silica particles resulted in nanocomposites with improved mechanical and thermal properties. DSC and SEM results indicated chemical bonding and strong interactions between the polymers and fillers.

The conducting polymer/mesoporous silica composites were synthesized by *in situ* polymerization of the corresponding monomers, such as phenylene butadiynylene [37], pyrrole [38,39,40,41], aniline [42,43] and diphenylamine [44] in the presence of mesoporous silica. Covalent graft of polyaniline onto the pores walls of SBA-15 was done by Sasidharan *et al*. [45]. They first synthesized monolayer N-propylaniline functionalized mesoporous silica SBA-15 by co-condensation of N-[3-(trimethoxysilyl)propyl]aniline and TEOS in the present of templates, and then the functionalized SBA-15 was contacted with aniline gas and polymerization occurred in the presence of ammonium peroxodisulfate as oxidant. The grafted polyaniline showed an increased electrical conductivity, compared to non-grafted composites.

Preparation of the conjugated polymer PPV inside the channels of the mesoporous silica MCM-41 was described by Pattantyus-Abraham *et al*. [46] .In this method, MCM-41 was first converted to a basic form by deprotonating its surface hydroxy groups with a nonaqueous base (tetrabutylammonium hydroxide). The *in situ* polymerization was performed by the transfer of the basic solid into the solution of xylylene bis(tetrahydrothiophenium chloride).

A poly(lactic acid)-coated mesoporous silica nanosphere (PLA-MSN) was prepared by Radu *et al*. [47]. In this method, a mercaptopropyl-functionalized mesoporous silica (thiol-MSN) was first synthesized, and then the exterior surface of thiol-MSN was grafted by 1,5,6-epoxyhexyltriethoxysilane. After the grafted epoxyhexyl groups were converted to 5,6-hydroxyhexyl groups by acid, L-Lactide together with a catalyst (tin(II) 2-ethylhexanoate) was added, and polymerization occurred, generating PLA-MSN. PLA-MSN can serve as a fluorescence probe for selective detection of amino-containing neurotransmitters under physiological conditions and can regulate the penetration of molecules in and out of the nanoscale pores by utilizing the PLA layer as a gatekeeper [47].

Dental composites composed of mesoporous silica and resin was prepared by Praveen *et al*. [48]. The composites were prepared by mixing bisphenyl A glycidyl dimethacrylateand, triethylene glycol dimethacrylate and mesoporous fillers. Camphorquinone and 2-(dimethylamino) ethyl methacrylate were used as photoinitiator and accelerator, respectively. The mesoporous fillers can improve the mechanical and aging properties of experimental dental composites. A rubbery epoxy polymer/mesoporous silica composite was synthesized by Jiao *et al*. [49,50]. The composites were synthesized by mixture of mesoporous silica with epoxy resin and curing agents (*α,ω*-polyoxypropylene diamine), and then reacted under 50 or 125 °C. It was demonstrated that an ordered porous network with animopropyl functional groups was essential for polymer reinforcement, and that larger framework pores were superior to those provided by smaller pore derivatives, most likely because of more efficient polymer impregnation of the particle mesopores [49,50]. 

Lin *et al*. reported polyimide (PI)/mesoporous silica composite films prepared by condensation polymerization of dianhydride and diamine with silylated mesoporous silica particles, followed with thermal imidization [51]. Min *et al*. synthesized PI/SBA-15 composites [52]. In this method, different SBA-15 powders modification by octyl or amino were used. The composite films containing SBA-15 modified with amino showed an increased in the tensile strength and elongation compared with those with octyl and pristine SBA-15. The dielectric constant was significantly reduced with increasing SBA-15 modified with amino groups [52]. The same composites were also prepared by Lin *et al.* [53], and the low dielectric constant was also observed for the PI filled with SBA-15.

A hybrid structure based on vinyl-functionalized MCM-48 and PS was prepared through *in situ* polymerization of styrene monomer [54]. The results indicated that the inorganic/organic nanocomposites coupled at the nanometer level when the monomers were polymerized with the intrapore grafted vinyl groups. A considerable improvement in Young's modulus and tensile strength were observed in the composites. The PS/MCM-41 [55] and Polyethylene/MCM-41 [56,57] composites were also prepared using *in situ* polymerization.

Many studies have focused on synthesis of ordered mesoporous silica/poly(*N*-isopropylacrylamide) (PNIPAAm) copolymer composites [58,59]. The composites were formed by co-condensation of TEOS and PNIPAAm-*co*-poly(3-methacryloxypropyltrimethoxysilane) in the presence of surfactants [58]. Magnetic mesoporous silica/PNIPAAm composites were also prepared recently [60,61]. These composites could be applied in targeting by magnetic field and controlled release of drugs by temperature change.

A pH-responsive core-shell mesoporous silica/ [poly(methacrylic acid-*co*-vinyl triethoxylsilane] sphere was synthesized by Gao *et al.* [62]. The poly(methacrylic acid-*co*-vinyl triethoxylsilane) was first synthesized via a copolymerization of methacrylic acid and vinyl triethoxylsilane monomer. This polymer was grafted onto the mesoporous silica via reaction of vinyl triethoxylsilane with Si-OH groups on the mesoporous silicas.

Chu *et al*. reported a bifunctional ordered mesoporous TiO_2_-SiO_2_-polymer nanocomposite synthesized by the direct triblockcopolymer-templating route using TEOS as a silica source, tetraisopropyl titanate as a titanium source, phenol and formaldehyde as polymer sources and Pluronic F127 as a template [63]. In this material, the pore walls are composed of polymer, silica and Ti species, and the organic polymers and inorganic solids are homogeneously dispersed inside the pore wall.

### 3.3. Surface-Initiated Polymerization

The grafting strategies to construct the polymer/mesoporous silica composite are of great interesting. Generally, two routes are used to graft polymer chains on the surface of the mesoporous silica, e.g., “grafting-to” and “grafting-from” technique [1,2]. The “grafting to” method refers to the grafting of polymers onto inorganic particles. This method usually generates nongrafted chains. Additionally, the grafted polymer chains prevent attachment of the next ones and then limit the graft density [1]. The “grafting-from” technique refers to polymer chains growth from the surface of the inorganic supports. This method is feasible to design polymer/mesoporous silica composite with covalent interaction between polymer and inorganic fillers.

**Figure 2:**
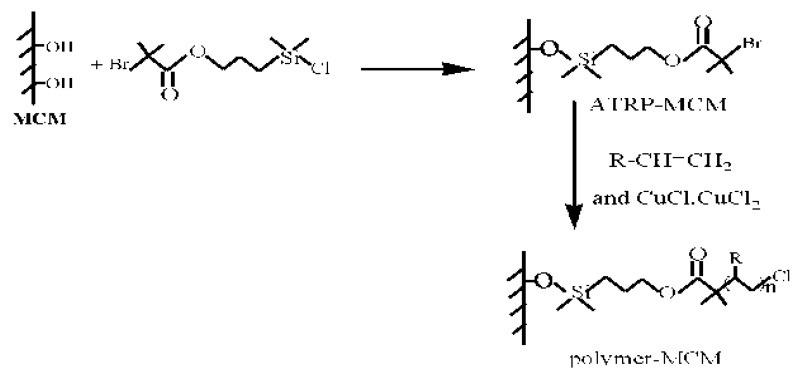
ATRP method for grafting polymer onto the mesoporous silica.

A stimuli-responsive mesoporous material was synthesized via surface-initiated polymerization (“grafting from” technique) [64,65]. The mesoporous silica was first covalently grafted to initiator, 1-(trichlorosilyl)-2-(*m*/*p*-(chloromethyl)phenyl)ethane. After addition of monomer NiPAAm, CuCl and bipydine, an atom transfer radical polymerization (ATRP) were performed on the surface of the mesoporous silica (Figure 2). The ATRP has also been applied to synthesize PS [66], polyacrylonitrile [66], poly(2-(dimethylamino)ethyl methacrylate) [66], PMMA [67,68] and peptide [69]/mesoporous silica composites.

**Figure 3:**
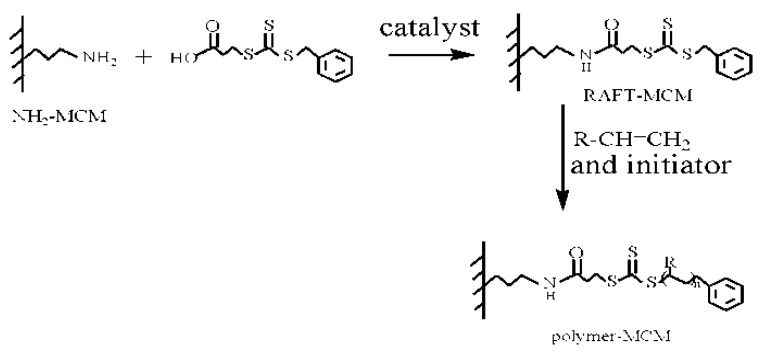
RAFT method for grafting polymer onto the mesoporous silica.

The polymer/mesoporous silica composites were also synthesized via a surface-initiated living radical polymerization with a reversible addition–fragmentation chain transfer (RAFT) reaction (Figure 3). A series of PNIPAAm-coated mesoporous silica nanoparticles [70,71,72] and PS shell/mesoporous silica nanoparticle cores [73] nanospheres were synthesized.

**Figure 4:**
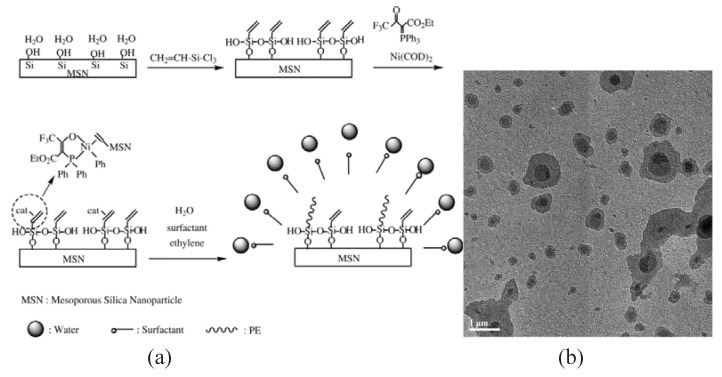
(a) The schematic illustration for emulsion polymerization of ethylene from MSN, (b) The TEM of the resulting PE/mesoporous silica composites. Reproduced with permission from [74]. Copyright 2009 John Wiley & Sons, Inc.

We recently reported on emulsion polymerization of ethylene from vinyl functionalized mesoporous silica nanoparticles (V-MSNs) [74]. V-MSNs, with the relative surface coverage of the vinyl monolayers 74%, were synthesized via deposition of vinyl monolayers on the pore walls (Figure 4). These V-MSNs with high surface coverage are desirable for anchoring metal catalysts (A fluorinated P-O-chelated nickel catalyst in this case) onto the nanopores for ethylene aqueous polymerization due to their advantages: high surface coverage with organic groups obviating the destructive interaction of reactive catalysts with silanol groups (Si-OH) lining the framework pore walls; high density of vinyl groups making catalysts easy coordinate to the vinyl groups; hydrophobic surface contributed by vinyl groups preventing catalysts from poison by water. PE chains grew from the pores of V-MSNs, formation of stable nanocomposite lattices with solid content up to 17.3%. Our method resulted in V-MSNs well-dispersed in the PE matrix. Especially, because of a strong interaction between PE and nanoparticles, a stable V-MSNs core/PE shell structure was formed upon thermal treatment above melting temperature of the PE (Figure 4b).

## 4. Other Methods

Some polymer/mesoporous silica composites can be synthesized by sol-gel method. Fujiwara *et al*. reported polymer sulfonate/mesoporous silica composites via so-gel method [75]. In this method, the polymer sulfonate (Nation and poly(sodium-styrenesulfonate)), TEOS and surfactants were simply mixed in alkaline aqueous solution to generated a composite. In this composite, organic polymers are incorporated in the framework of mesoporous silica. Madhugiri *et al*. electrospinned MEH-PPV/SBA-15 composite nanofibers using a dual syringe method [76]. During this electrospinning process, two syringes one containing MEH-PPV solution and the other containing SBA-15 gel were subjected to a voltage of 20 kV. An interesting result is that fluorescence of MEH-PPV was blue shifted in the composites.

## 5. Summary and Outline

This review summarized the recent developments in the methods to synthesize polymer/mesoporous silica nanocomposites. Most of the composites were synthesized via blending and *in situ* polymerization methods. Although these methods have been studied extensively, to further development new method for fabrication of mesoporous silica-based nanocomposites is still a hot topic. 

The mesoporous silica-based nanocomposites are relatively new materials, which have only received attention in recent years, and many properties of these materials have not been disclosed. The study of the unique properties of these materials has received much attention and an increasing amount of papers has been published in recent year. Especially, the colloidal polymer/mesoporous silica nanospheres can load drugs and easy transfer cross the living cells [77]. The grafted polymer can be designed to control the loaded drug release in the cells. These features make colloidal polymer/mesoporous silica nanospheres an excellent candidate for next-generation drug carriers. 
